# Serum value of fasting C-peptide (FC-P), fasting insulin (FIns), and glycated hemoglobin (HbA1c) after dynamic blood glucose monitoring-guided personalised nutrition and insulin pump therapy for type II diabetes mellitus

**DOI:** 10.5937/jomb0-55738

**Published:** 2025-09-05

**Authors:** Qing Zhou, Li Zou, Yan Gao, Hua Ma, Yaping Guo, Guohong Zhu

**Affiliations:** 1 T aizhou People's Hospital, Department of Endocrinology, T aizhou, Jiangsu Province, China

**Keywords:** serum value of fasting C-peptide (FC-P), fasting insulin (FIns), glycated haemoglobin (HbA1c), T2DM, continuous glucose monitoring, quality circle control nursing model, individualised nutrition, insulin pump therapy, serumska vrednost C-peptida natašte (FC-P), insulina natašte (FIns), glikoziliranog hemoglobina (HbA1c), T2DM, kontinuirano praćenje glukoze, model nege kontrolnog kruga kvaliteta, individualizovana ishrana, terapija insulinskom pumpom

## Abstract

**Background:**

Type 2 diabetes mellitus (T2DM) is a chronic metabolic disorder characterised by impaired glucose metabolism, which necessitates comprehensive management of blood glucose (BG), blood pressure, and lipid profiles. This study aimed to evaluate the clinical effects of individualised nutrition and insulin pump therapy, guided by continuous glucose monitoring (CGM) and the Quality Circle Control (QCC) nursing model, on various biomarkers in T2DM patients, including fasting C-peptide (FC-P), fasting plasma glucose (FPG), 2-hour postprandial glucose (2hPG), fasting insulin (FIns), and glycated haemoglobin (HbA1c).

**Methods:**

Eighty T2DM patients treated at our hospital were enrolled in the study between January 2023 and January 2024. Patients were assigned to either the experimental group (EG), which received individualised nutrition and insulin pump therapy supported by CGM and the QCC nursing model, or the regular group (RG), which received standard care. Differences in BG control, insulin usage, CGM system performance (including downtime and anomaly rates), and patient satisfaction were compared between the two groups.

**Results:**

The EG demonstrated significant improvements in FC-P, FPG, 2hPG, FIns, and HbA1c levels compared to the RG (P<0.05). Specifically, the EG showed more rapid achievement of BG targets, reduced glucose variability, lower insulin usage, and decreased CGM system anomalies.

**Conclusions:**

The QCC nursing model, when integrated with individualised nutrition and insulin pump therapy guided by CGM, significantly enhances blood glucose control, optimises insulin therapy, and improves patient outcomes, including dietary habits, quality of life, and reduction in hypoglycemic events. This model shows promise as an effective strategy for managing T2DM and warrants further adoption in clinical practice.

## Abstract (sr)

**Uvod:**

Dijabetes melitus tipa 2 (T2DM) je hronični metabolički poremećaj karakterisan oštećenim metabolizmom glukoze, što zahteva sveobuhvatno upravljanje nivoima glukoze u krvi (BG), krvnim pritiskom i lipidnim profilima. Ova studija imala je za cilj da proceni kliničke efekte individualizovane ishrane i terapije insulinskom pumpom, vođene kontinuiranim praćenjem glukoze (CGM) i modelom nege Kontrolnog kruga kvaliteta (QCC), na različite biomarkere kod pacijenata sa T2DM, uključujući C-peptid natašte (FC-P), glukozu u plazmi natašte (FPG), glukozu 2 sata nakon obroka (2hPG), insulin natašte (FIns) i glikozilirani hemoglobin (HbA1c).

**Metode:**

U studiju je uključeno osamdeset pacijenata sa T2DM, lečenih u našoj bolnici između januara 2023. i januara 2024. godine. Pacijenti su podeljeni u eksperimen-talnu grupu (EG), koja je primala individualizovanu ishranu i terapiju insulinskom pumpom uz podršku CGM-a i QCC modela nege, i redovnu grupu (RG), koja je primala stan-dardnu negu. Vršena su upoređivanja razlike između dve grupe u kontroli BG, upotrebi insulina, performansama CGM sistema (uključujući prekide rada i stope anomalija) i zadovoljstvu pacijenata.

**Rezultati:**

EG je pokazala značajna poboljšanja u nivoima FC-P, FPG, 2hPG, FIns i HbA1c u poređenju sa RG (P<0,05). Konkretno, EG je brže postizala ciljne vrednosti BG, smanjila varijabilnost glukoze, smanjila upotrebu insulina i smanjila anomalije CGM sistema (P<0,05).

**Zaključak:**

Model QCC nege, kada se integriše individualizovanom ishranom i terapijom insulinskom pumpom vođenom CGM-om, značajno poboljšava kontrolu glukoze u krvi, optimizuje insulinsku terapiju i unapređuje ishode pacijenata, uključujući prehrambene navike, kvalitet života i smanjenje hipoglikemijskih epizoda. Ovaj model pokazuje potencijal kao efikasna strategija za upravljanje T2DM i zaslužuje širu primenu u kliničkoj praksi.

## Introduction

Type 2 diabetes mellitus (T2DM) is a chronic metabolic disease characterised by insulin resistance and β-cell dysfunction, causing sustained elevation of blood glucose (BG) levels. Globally, the incidence of T2DM is rapidly increasing, posing a great challenge in the field of public health in China. There were 536.6 million adults worldwide (10.5%) with T2DM, projected to increase to 783.2 million (12.2%) by 2045 [1]. A survey found a drastic increase in diabetes-related deaths among urban and rural residents in China from 1987 to 2019, with urban areas consistently exhibiting higher mortality rates than rural areas over the past 30 years. However, due to a rapid rise in rural diabetes mortality rates in recent years, this urban-rural disparity has gradually narrowed [2]. Recent studies reported a diabetes prevalence of 11% among Chinese adults [3]. Individuals with T2DM not only face risks of serious complications such as cardiovascular disease, kidney disease, retinopathy, and cancer [4] [5], but also impose substantial economic burdens on the healthcare system and society as a whole.

In the daily management of T2DM, continuous glucose monitoring (CGM) technology is increasingly widespread. It is crucial for devising personalised treatment plans and lifestyle choices and providing patients with essential information for BG control. In clinical practice, haemoglobin A1C (HbA1c) measurement has become the gold standard for glucose monitoring and diabetes management, reflecting average BG levels over the past 2 to 3 months [6]. However, HbA1c measurements do not provide detailed information on key clinical indicators such as glycemic variability (GV) and frequency of hyperglycemic or hypoglycemic events. Additionally, the reliability of HbA1c measurements may be questioned in patients with specific complications such as anaemia, hemoglobinopathies, or severe decompensated liver disease.

Nevertheless, the limitations of HbA1c can be addressed through self-monitoring of BG (SMBG) and CGM technologies. CGM has become an essential tool for daily BG monitoring in diabetes patients since its market introduction. Compared to traditional fingertip blood sampling methods, CGM provides continuous, real-time glucose data, helping patients better understand their glucose fluctuation patterns [7]. CGM systems include real-time CGM (rt-CGM) and intermittently scanned CGM (is-CGM). These systems offer healthcare providers comprehensive glucose profiles, enabling more accurate assessment of patients' glucose control status and timely detection and prevention of hypoglycemic and hyperglycemic events. As advanced insulin delivery systems, insulin pumps mimic the natural insulin secretion mechanism of the pancreas by continuously delivering insulin subcutaneously, providing either continuous or on-demand insulin delivery [8]. The *American Association of Clinical Endocrinologists *recommends CGM for all diabetes patients undergoing intensive insulin therapy, defined as those using insulin pumps or requiring three or more insulin injections daily [9]. Insulin pump use has been demonstrated to improve BG control, reduce hypoglycemic events, and potentially preserve b-cell function in patients with T2DM [10].

Due to the severe adverse events associated with BG abnormalities in patients with T2DM and the incurable nature of the disease, careful attention to diet is crucial for these individuals. Personalised nutrition therapy is widely recommended in the daily care of T2DM patients. This approach tailors dietary plans based on individualised BG data, dietary habits, and lifestyle to achieve optimal glycemic control targets. In diabetes management, the quality circle control (QCC) nursing Model emphasises patient-centred care, encouraging active patient involvement in selfmanagement while providing essential education and support. This model has been proven effective in enhancing patients' ability to self-manage their health, reducing complications associated with the disease, and significantly improving their quality of life [11] [12]. However, its specific effects on diabetes patient care have not been extensively studied. Therefore, this work aimed to investigate the clinical effects of individualised nutrition and insulin pump therapy guided by CGM based on the QCC nursing Model in T2DM patients. The goal was to provide robust evidence supporting the application of this comprehensive management strategy in T2DM patients, offering new therapeutic options for clinical practice and establishing scientific foundations for long-term diabetes management.

## Materials and methods

### General information

This work enrolled 80 patients with T2DM treated at our institution from 2023 to 2024. Patients were randomly assigned to either the research group (EG) or the regular group (RG), with 40 patients in each group. The institutional ethics committee approved the study protocol, and all participating patients and their families provided informed consent.

Inclusion criteria: patients diagnosed with diabetes according to the *Diagnosis and Treatment Guidelines for T2DM (2019 edition)* [13]; HbA1c ≥6.5%; first-time treatment for diagnosed diabetes; patients who signed informed consent forms.

Exclusion criteria: non-T2DM patients; patients with severe organ dysfunction; patients with psychiatric disorders affecting their ability to participate; patients unable to articulate their feelings; patients with concurrent diseases affecting BG levels.

### Intervention methodologies

All patients received treatment according to medical recommendations, including medication for managing T2DM.

EG underwent individualised nutrition and insulin pump therapy guided by CGM based on the QCC nursing Model. Here are the details of CGM guidance:

### (1) CGM Guidance

Patients were monitored using a subcutaneous dynamic glucose monitoring system (San Medino), which collects BG values every 10 seconds and calculates average glucose values every 3 minutes, storing data retrospectively for up to 72 hours to review glucose fluctuation trends. The BG monitoring range was set between 1.7 and 25.0 mmol/L. Thresholds for BG control were established based on individual patient conditions, triggering automatic alerts from the glucose meter when these thresholds were exceeded.

### (2) QCC nursing:

I. Establishment and training of QCC nursing team: a QCC nursing team of 7 members was established. This team included 1 team leader (an endocrinology nurse with over 15 years of experience), 2 senior nurses, 3 junior nurses, and 1 nurse. The training program spanned six months, during which all team members received theoretical knowledge and technical training. The team leader was responsible for planning and implementing the QCC nursing. II. Theme selection: during the first three months of training, themes were selected at each meeting and discussed about the chosen topics. During the last three months of training, team members discussed their own chosen topics. III. Periodic evaluation: the team periodically reviewed the nursing process, identified issues, and optimised strategies within the QCC nursing model to enhance effectiveness. IV. Optimisation of QCC nursing for T2DM patient management: educating patients on the principles and functions of the CGM system was primarily the responsibility of the QCC nursing team. Nurses may lack sufficient understanding of the CGM system, which could lead to delays in resolving system malfunctions and potentially cause psychological stress to patients. Therefore, enhancing training on the CGM system was necessary to promptly address issues such as CGM downtime and alerts optimising care strategies for T2DM patients. Strengthening dietary management involved tailoring individualised nutrition plans based on patient conditions, with a daily intake of 25-40 Kcal/kg. The nutritional components are presented in Table 1.

**Table 1:** Daily nutrition matching.

Type	Proportion
Fat	25%-35%
Carbon water	45%-60%
Protein	15-20%

Exercise for at least 150 minutes per week. Combining the CGM system during exercise to prevent hypoglycemic reactions.

In RG, patients received standard care for T2DM. The number of healthcare team members, their qualifications, and years of service were comparable to those in the QCC nursing group, but they did not undergo QCC nursing training. Upon admission, patients were given a T2DM health guidance manual to disseminate knowledge on T2DM health and standardise lifestyle habits and daily dietary practices. Guidance included meal timing, medication schedules, portion control, and appropriate physical exercise to maintain optimal body weight. Following admission, patients received CGM guidance, performing fingertip BG monitoring before breakfast, lunch, and dinner. BG readings were input into the CGM system, and insulin pumps administered insulin (regular insulin) based on glucose levels for treatment.

### Observation indicators

### (1) Main results

a. Patients were evaluated based on insulin levels and BG normalisation. Treatment was deemed significantly effective if insulin levels and BG normalised, leading to the disappearance of T2DM symptoms. Treatment was considered effective if insulin levels and BG improved by over 80%. Treatment was classified as ineffective if there was no significant improvement in insulin levels and BG compared to pre-treatment.

b. Patient satisfaction between the two groups was assessed using a hospital-developed CGM-guided patient satisfaction questionnaire based on the QCC nursing model. Evaluation criteria included medical and nursing responsibility, operational skills, service attitude, and safety management, with a maximum score of 100 points. Scores were categorised as: very satisfied (≥95 points), satisfied (≥80 and <95 points), and unsatisfied (<80 points).

### (2) Secondary results

a. Fasting C-peptide (FC-P), fasting plasma glucose (FPG), 2-hour postprandial glucose (2 hPG), fasting insulin (FIns), and glycated haemoglobin (HbA1c) levels were compared before and after treatment in both groups. FPG, 2 hPG, and HbA1c levels were measured using a glucometer.

b. Insulin function was compared between the two groups using the modified HOMA equation to calculate patient insulin function.

c. The time to achieve BG targets, changes in BG values, and insulin pump usage were compared between the two groups.

d. The anomalies related to the CGM system during patient care, including instances of CGM system downtime, alarm frequency, and skin allergies, were compared.

### Statistical analysis

The data were processed using SPSS 26.0. Continuous variables were denoted as mean±standard deviation and inter-group analyses were conducted using independent samples t-tests. Categorical variables were presented as (n(%)) and analysed between groups using the chi-square (χ^2^) test. *P*<0.05 was utilised to determine statistical significance.

## Results

### Comparison of general information

Statistical analysis of general patient information for both groups is detailed in Table 2. The baseline characteristics differed inconsiderably between groups (*P*< 0.05).

**Table 2:** General information.

General information	RG (n=40)	EG (n=40)	χ^2^/t	P
Gender			0.05	0.823
Male	19	18		
Female	21	22		
Age	55.72±14.69	58.64±13.75	0.821	0.781
BMI	24.84±3.17	25.16±3.84	0.914	0.793
Disease course	6.01±1.12	5.97±0.95	1.023	0.649

### Clinical efficacy of two nursing methods

**Figure 1:**
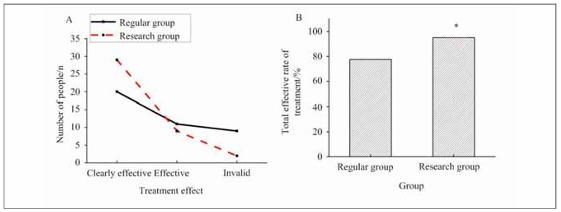
Comparison of clinical efficacy between two nursing approaches. (A: number of patients with treatment effect; B: overall treatment efficacy)<br>Note: *P<0.05 vs. RG.

The study analysed the treatment efficacy of patients (Figure 1). In EG, 29 patients showed significant effectiveness of treatment, which was markedly superior to the 20 patients in RG who showed significant effectiveness. Furthermore, the overall treatment effectiveness rate was analysed between the two groups. The treatment effectiveness rate in RG was 77.5%, whereas in EG, it was 95.0%, notably superior to that in RG (*P*=0.043).

### Comparison of patient satisfaction between two nursing methods

**Figure 2:**
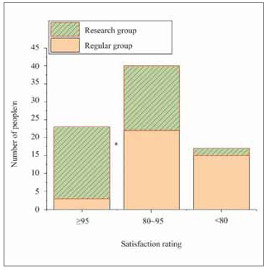
Comparison of patient satisfaction between two nursing approaches.<br>Note: *P<0.05 vs. RG.

The satisfaction with nursing care methods between the two groups of patients was statistically analysed, with results shown in Figure 2. The number of patients in EG who reported being »very satisfied« with the nursing care was substantially superior to RG (*P*<0.05).

### BG indicators

**Figure 3:**
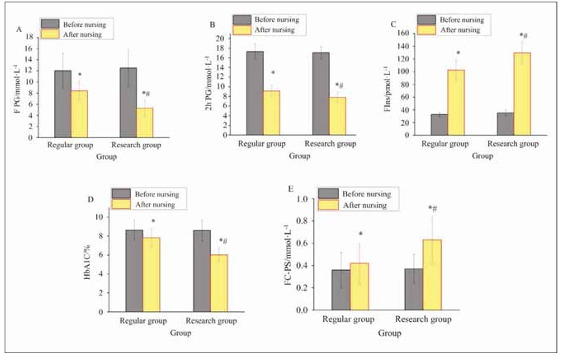
Comparison of BG indicators before and after nursing. (A: FPG; B: 2h PG; C: Fins; D: HbA1c; E: FC-PS)<br>Note: *P<0.05 vs. before nursing, #*P*<0.05 vs. RG.

A comparison of the impact of two nursing methods on BG indicators in T2DM patients is presented in Figure 3. Before the nursing intervention, BG indicators (FPG, 2h PG, FIns, HbA1c, FC-PS) differed slightly between groups (*P*>0.05). However, after nursing, great improvements were observed in the BG indicators compared to before intervention in both groups (*P*<0.05). Specifically, patients in EG showed more drastic reductions in FPG, 2h PG, and HbA1c versus RG (*P*<0.05), while increases in FIns and FC-PS were also more pronounced in EG than in RG (*P*<0.05), demonstrating substantial differences.

### Insulin function

**Figure 4:**
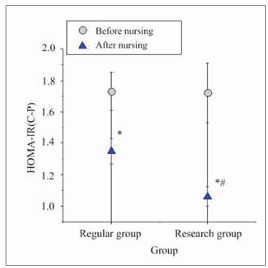
Comparison of insulin function before and after nursing in two groups of patients.<br>Note: *P<0.05 vs. before nursing, #*P*<0.05 vs. RG.

A comparison of the impact of two nursing approaches on insulin function in T2DM patients is illustrated in Figure 4. Prior to nursing intervention, no notable differences existed in HOMA-IR (C-P) values between groups (*P*>0.05). After the intervention, both groups experienced drastic reductions in HOMA-IR (C-P) values relative to their baseline levels (*P*<0.05). Specifically, patients in EG demonstrated more pronounced decreases in HOMA-IR (C-P) values relative to those in RG (*P*<0.05), highlighting marked differences.

### Comparison of BG target time, changes in BG levels, and insulin dosage

**Figure 5:**
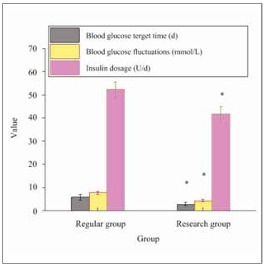
Comparison of glycemic target achievement time, glycemic changes, and insulin usage after nursing in two groups of patients.<br>Note: *P<0.05 vs. RG.

Observation of the clinical outcomes of the two nursing approaches (Figure 5) revealed that relative to RG, the nursing approach in EG greatly shortened the time to achieve BG targets (5.84±1.26 vs. 2.93±0.84, *P*<0.05), reduced glycemic variability (7.83±0.62 vs. 4.39±0.57, *P*<0.05), and decreased insulin usage (52.28±3.19 vs. 41.72± 3.25, *P*<0.05).

### Abnormal occurrence of CGM system during nursing care for two groups of patients

**Figure 6:**
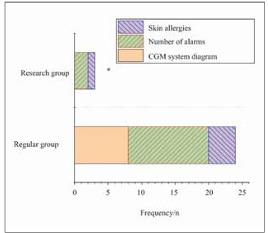
Comparison of CGM system abnormalities during nursing.<br>Note: *P<0.05 vs. RG.

Under equal nursing durations, adverse events related to CGM system usage were compared between the two groups of patients (Figure 6). EG exhibited notably lower CGM system interruptions, alarm occurrences, and skin allergies during nursing versus RG (*P*<0.05), indicating a considerable difference.

## Discussion

Research using systematic dynamic modelling has examined the projected growth of the diabetes population in China, predicting that by 2050, annual deaths among diabetic patients will reach 18.255 million [14]. Several factors contribute to this alarming increase in diabetes-related mortality, with poor glycemic control and complications such as cardiovascular disease, neurological disorders, and infections being the primary causes [15] [16] [17] [18]. The standard clinical measures for managing Type 2 Diabetes Mellitus (T2DM) typically include fingertip blood glucose (BG) testing and insulin therapy. However, patients often face significant challenges with hypoglycemia, leading to risks such as falls, accidents, and delayed wound healing, particularly after infections or surgeries. These factors emphasise the importance of continuous glucose monitoring (CGM) and carefully managed meal timings for optimal diabetes control.

Recent advances in CGM systems, including real-time continuous glucose monitoring (rt-CGM), have proven more effective in providing tighter BG control and better-detecting fluctuations in glucose levels [19] [20]. These systems allow for 24-hour monitoring, detecting early signs of glucose abnormalities that are otherwise difficult to identify with conventional BG testing. The early detection of these changes is vital, as it allows for timely intervention to prevent both acute and long-term complications.

In addition to CGM's role in improving glucose control, serum biomarkers have become essential in evaluating the effectiveness of diabetes management. Key markers such as fasting plasma glucose (FPG), glycated haemoglobin (HbA1c), fasting insulin (FIns), and C-peptide (FC-P) levels serve as critical indicators of glycemic control and pancreatic β-cell function. Research has demonstrated that using rt-CGM systems in conjunction with these biomarkers significantly improves glycemic management and insulin sensitivity in T2DM patients. For instance, in a study of 4,154 T1DM and 13,623 T2DM patients, rt-CGM systems resulted in an 18.2 mg/dL reduction in average BG levels in T2DM patients [21] [22] [23] [24] [25]. Similarly, a 1.9-year follow-up study showed that CGM-guided treatment reduced HbA1c levels from 7.21% to 7.00%, highlighting the clinical advantages of continuous monitoring to optimise BG control over time [26].

The effectiveness of the CGM system extends beyond just glucose levels. By assessing insulin function via the Homeostasis Model Assessment of Insulin Resistance (HOMA-IR), studies have shown a significant improvement in insulin sensitivity when CGM is integrated into diabetes management. Reductions in HOMA-IR values, which reflect lower insulin resistance, suggest that CGM not only aids in controlling BG but also enhances pancreatic b-cell function, which is crucial for long-term diabetes management [27]. Moreover, these reductions are further supported by improvements in serum markers like C-peptide levels, indicating better insulin secretion and β-cell recovery.

Furthermore, CGM has been shown to capture more dynamic glucose fluctuations than traditional BG testing, offering insights into the continuous nature of glucose regulation. CGM systems are especially beneficial in intensive care and neonatal settings, where precise glucose control is critical for preventing severe complications [27]. The integration of CGM with insulin pump therapy, tailored nutrition plans, and real-time feedback can thus lead to more effective management of T2DM, as evidenced by improved glucose control markers such as FPG, 2hour postprandial glucose (2h PG), HbA1c, and insulin levels in patients receiving personalised treatment strategies [28] [29] [30] [31] [32] [20].

Although the QCC nursing model has been shown to improve patient satisfaction in other clinical areas [21] [22], this study focused on the clinical benefits of using CGM-guided treatment to improve BG control and reduce complications associated with T2DM. The individualised care facilitated by CGM and insulin pump therapy is crucial for managing the complex, multifaceted nature of T2DM. Studies have demonstrated that personalised treatment approaches, informed by CGM data and serum markers, lead to more favourable clinical outcomes, including enhanced insulin sensitivity and better overall glycemic control.

In terms of clinical outcomes, this study demonstrated that patients treated under CGM guidance had reduced glucose variability, quicker achievement of target BG levels, and improved insulin utilisation compared to traditional approaches. These findings underscore the importance of incorporating advanced glucose monitoring systems into routine clinical practice, particularly for T2DM patients who struggle with glycemic variability and insulin resistance.

Despite these improvements, it is important to recognise potential challenges related to CGM system use, such as downtime, device malfunctions, and skin allergies. Addressing these issues through better training and optimisation of CGM management practices is essential to ensure that the benefits of CGM are maximised in everyday clinical settings. Future studies could further explore how to refine CGMbased management strategies and address any barriers to their widespread implementation.

The study reinforces the significant role of CGM in improving glycemic control in T2DM patients, alongside improvements in serum markers that reflect insulin resistance and pancreatic function. The combination of real-time glucose monitoring, insulin therapy, and personalised treatment approaches is promising for long-term diabetes management. However, attention should be paid to potential technical issues associated with CGM systems, and ongoing efforts should focus on optimising their use in clinical practice.

## Conclusion

The study results indicate that personalised nutrition and insulin pump therapy guided by the QCC nursing model and CGM greatly improve patients' BG parameters, enhance treatment efficiency, and increase nursing satisfaction. Furthermore, this approach notably improves the HOMA-IR (C-P) index, reduces the time to achieve target BG levels, decreases BG variability, and lowers insulin requirements. CGM system interruptions, alarms, and skin allergies during nursing care are significantly reduced, potentially improving long-term patient prognosis and quality of life. Despite potential challenges associated with CGM system use, such as devicerelated discomfort and operational complexity, effective resolution of these issues through appropriate training and support ensures patients can use CGM systems safely and comfortably for BG monitoring.

## Dodatak

### Conflict of interest statement

All the authors declare that they have no conflict of interest in this work.
